# A Glance at the Rise and Progress of Cell Theories

**Published:** 1870-08

**Authors:** I. N. Danforth

**Affiliations:** Chicago


A MONTHLY RECORD OF

*Medicine, Surgery, and the Collateral Sciences.*

EDITED BY J. ADAMS ALLEN, M.D., LL.D.; AND WALTER HAY, M.D.

---

VOL. XXVII. — AUGUST, 1870. — No. 8.

---

### Original Communications.

ARTICLE I.—*A Glance at the Rise and Progress of Cell Theories.* By I. N. DANFORTH, M.D., Chicago.

From an early period in the history of medicine, Physiologists and Anatomists seem to have realized that there *must* be some ultimate atom; some anatomical unit, from which the parts and organs of the body are derived, and by which they are maintained. But, although we are in possession of pretty reliable evidence that the magnifying properties of lenses were known to the ancients, we have no reason to believe that they were ever used for purposes of scientific investigation. Indeed, the specimens found in course of the excavations at Herculaneum and Pompeii are so rude and coarse as to be utterly useless except as mere toys. It is not therefore surprising that their knowledge of ultimate structure was next to nothing. Fallopius, about the year 1523, described elementary structure as consisting of bone, cartilage, fat, flesh, nerve, ligament, tendon, membrane, vein, artery, nails, hair, and skin—which, at the very best, was but a very faulty and incorrect classification of the tissues as they appear to the naked eye.

Although the compound microscope was invented about the year 1590 by the Jansens of Holland, (the Italians also claim priority) nearly a century elapsed before it was used to any extent in the examination of the animal tissues. Borellus, of Pisa, about 1656, seems to have been a "microscopist" of some note; but, like quite too many modern observers, he was afflicted with a "theory," and his observations were terribly distorted in support thereof. According to him, diseases were caused by animalculæ in the blood and tissues; consequently *his* peculiar mission was to *find* animalculæ—and of course he succeeded. He describes pus corpuscles as animalcules—and says that he has actually *seen* them depositing their ova! Did he not see the process of cell development by budding, and misapprehend its import?

On the authority of Boerhaave, Swammerdam discovered the blood globule in 1658. Towards the close of the seventeenth century, considerable progress seems to have been made in microscopic investigation, and a somewhat correct appreciation of the importance of this instrument seems to have been developed. Malpighi, in 1661–5, published accounts of the minute structure of the lungs, kidneys, spleen and liver; Hooke, in 1667, described the *cellular* structure of plants, and, in 1670, Malpighi made still further contributions in the same direction. The latter observer also conferred upon the cell the name of "utricle," thus taking the first step in establishing the confused nomenclature with which the subject of cytology is harassed and burdened to-day. But, to atone for that, Malpighi taught the very important doctrine that the cell or "utricle" is the seat of inherent vitality; that it is, of itself, an independent entity. Leeuwenhœck, in 1687, demonstrated the capillaries, and discovered the spermatozoa, ascribing to them their correct and proper function.

But no attempt seems to have been made at a systematic construction of the tissues of the body from a constant and ultimate element, until Haller, about 1757, promulgated his "fibre" theory. According to him, the tissues consist of two elements, namely, "fibre" and "organized concrete," whereof the former is the most important in that it is the seat of "irritability"—"the cause for all the phenomena of life"—and, consequently, the ultimate or primary agent of the phenomena of life. "Organized concrete," on the other hand, is a "mere glue" (Tyson) or connective substance—and its functions are accordingly altogether physical. But this theory could not have been founded upon microscopic investigation, since he says that the fibre is invisible—distinguishable only by the "mind's eye." It is interesting, however, as an evidence of his belief in a *structural element*, uniform in appearance, and constantly present, which was the true seat of vital manifestations.

The theory of Wolf seems to have been a step backward rather than forward. He taught that the cell is a mere cavity, produced by the development ("differentiation") of the nutritive fluid, and not an independent entity; that this organization proceeded under the guiding influence of the *vis essentialis* or organizing power, resident in the nutritive fluid, thus transferring the seat of vitality from the cell to its pabulum or "blastema"—than which a greater error could hardly have been committed.

After the downfall of the "fibre theory," the globule asserted *its* claims to notoriety—and this was certainly a step in the advance, since it led *towards* truth, whereas the "fibre theory" led precisely in the opposite direction. From 1779 to 1839 the globule maintained its importance as an ultimate element. But it derived its chief importance from the labors of Milne-Edwards, whose work, published in 1823, was largely devoted to the elaboration of the "globular theory"—according to which the principal tissues of the body were constructed from globules, measuring from $\frac{1}{8000}$ to $\frac{1}{7500}$ of an inch in diameter. Meantime, we must not overlook two facts; first, that great confusion had arisen in the terms employed by different writers—the words *granule*, *globule* and *molecule* being interchangeably and carelessly used, without a really definite meaning being attached to either; and, secondly, that the instruments employed were necessarily imperfect and coarse, and almost totally wanting in many of the accessories at this day deemed indispensable for the successful prosecution of minute investigations. Hence, it is more than probable that the true cell was actually seen long before its announcement by Schleiden, although its importance was totally unappreciated.

But the history of cytology, as a systematic branch of study, really commences with the observations of Schleiden and Schwann in the year 1838—so long was it from the invention of the compound microscope, to its practical utilization in the broad field of physiological inquiry. The observations of Schleiden were chiefly confined to vegetable structure; he conceived and announced the theory that the cell is the structural unit, and that all plants are developed after a uniform method by means of cell multiplication. This theory "Schwann immediately extended to animal tissues," (Tyson), and the goal was reached! The importance of this announcement, in its bearings on physiology and pathology, can scarcely be overestimated; it was hardly, if at all, less momentous than the discovery of the circulation of the blood by Harvey. But, while Schleiden and Schwann took a long stride in the advance, they also fastened upon us a most egregious error, which the weight of their great authority served to perpetuate for years —namely, "free cell development," which is only another name for spontaneous generation. So late as 1857, that excellent scholar and writer, Peaslee, of New York (Histology, page 120), states that cells are developed "directly from the plasma, without the aid of a pre-existing cell." Intending to allude to this subject more at length in a subsequent paper, I content myself for the present by saying that I believe this doctrine to be utterly erroneous and dangerous. The nucleus, discovered by Brown, of Edinburgh, in 1833, was named, by Schleiden, the "cytoblast," or cell bud, and the hyaline fluid was by the same observer called the "blastema," or formative element of the tissues, which was but adding another rivet to the chain which binds us to the dead body of spontaneous generation. The word "blastema," however, is not to blame for this; it was *forced* into this unwilling service by Schleiden, having been frequently used by Hippocrates in a far different and more appropriate connection. It is somewhat uncertain whether the nucleolus was first discovered by Schleiden or Valentin; it is more than probable, however, that the merit of this discovery belongs to the former, although the term "nucleolus" was first applied by Schwann.

The discoveries of Henle (1841), who in general adopted the views of Schwann regarding primary cell development, chiefly relate to cell multiplication, and, on this subject, his statements have stood the test of time. He taught that cells multiply in three ways, namely: by segmentation, by endogenous cell development, and by "budding." The discovery of the last named mode of development was a most important addition to our knowledge, though destined to lie dormant for many subsequent years.

The observations of Martin Barry (1840) relate to the changes that occur in the ovum *post coitum;* he recognized the fact that the ovum is practically a cell, and carefully studied and accurately described the changes which take place therein during development. He also called attention to the "germinal vesicle," which he correctly regarded as the proper "nucleus," and the "germinal spot," which, with equal correctness, he regarded as the "nucleolus." But the chief merit of Barry seems to have been, that he traced the development of cells from pre-existing cells—or "parent centres"—thus striking the first vigorous blow at the then popular notion of "free cell development."

Prof. John Goodsir seems to have added but little to our knowledge regarding cell *structure;* but he clearly conceived and quite fully announced the theory concerning cell *action*, which of late years has been so vigorously enforced by Virchow. Briefly, this theory is as follows: first, the body is divided into innumerable islets or "centres" of nutrition; these nutritive centres consist essentially of groups of cells, and these cells are in some sort presided over by, and are tributary to one central or capital cell, the latter being the probable parent of the others. This, it will be observed, is scarcely more or less than Virchow's doctrine regarding "cell territories." Secondly, according to this writer, the nucleus is the true centre of nutrition; the active and essential part of the cell; and all nuclei or nutritive centres are derived from pre-existing nuclei, and are never originated by a so-called "blastema," or formative substance, by any power outside the nuclei, and in this he assuredly anticipated Virchow. I think it is undeniably true that, however much or little merit there may be in this theory, quite too much praise has been awarded Virchow, and quite too little to Goodsir therefor; but certainly Virchow himself has not failed to properly recognize and acknowledge Prof. Goodsir's labors.

In a future paper, I propose to examine the doctrines of Huxley, Virchow and Beale, somewhat at length, together with their bearings on the present status and tendency of "cell doctrines." In conclusion, I wish to acknowledge my indebtedness to the excellent little work of Dr. Tyson, of Philadelphia, "The Cell Doctrine,"* for very much of the material incorporated in the present article.

* For sale by W. B. Keen & Cooke, Chicago.

